# Metabolic reprogramming of glioblastoma cells by L-asparaginase sensitizes for apoptosis *in vitro* and *in vivo*

**DOI:** 10.18632/oncotarget.9257

**Published:** 2016-05-09

**Authors:** Georg Karpel-Massler, Doruntina Ramani, Chang Shu, Marc-Eric Halatsch, Mike-Andrew Westhoff, Jeffrey N. Bruce, Peter Canoll, Markus D. Siegelin

**Affiliations:** ^1^ Department of Pathology & Cell Biology, Columbia University Medical Center, New York, New York, United States of America; ^2^ Department of Neurological Surgery, Columbia University Medical Center, New York, New York, United States of America; ^3^ Department of Neurosurgery, Ulm University Medical Center, Ulm, Germany; ^4^ Department of Pediatrics and Adolescent Medicine, Ulm University Medical Center, Ulm, Germany

**Keywords:** apoptosis, L-asparaginase, ABT263, TRAIL, glioblastoma

## Abstract

Cancer cells display a variety of global metabolic changes, which aside from the glycolytic pathway largely involve amino acid metabolism. To ensure aggressive growth, tumor cells highly depend on amino acids, most notably due to their pivotal need of protein synthesis. In this study, we assessed the overall hypothesis that depletion of asparagine by *E. coli*-derived L-asparaginase might be a novel means for the therapy of one of the most recalcitrant neoplasms and for which no efficient treatment currently exists - glioblastoma (WHO grade IV). Our results suggest that certain glioma cell cultures are particularly susceptible to inhibition of proliferation by L-asparaginase, while others display a more resistant phenotype. In sensitive cells, L-asparaginase induces apoptosis with dissipation of mitochondrial membrane potential and activation of effector caspases. L-asparaginase-mediated apoptosis was accompanied by modulation of pro- and anti-apoptotic Bcl-2 family members, including Noxa, Mcl-1 and the deubiquitinase Usp9X. Given the impact of L-asparaginase on these molecules, we found that L-asparaginase potently overcomes resistance to both intrinsic apoptosis induced by the Bcl-2/Bcl-xL inhibitor, ABT263, and extrinsic apoptosis mediated by TRAIL even in glioma cells that are resistant towards L-asparaginase single treatment. RNA interference studies showed that Usp9X, Mcl-1, Noxa and Bax/Bak are involved in ABT263/L-asparaginase-mediated cell death. *In vivo*, combined treatment with ABT263 and L-asparaginase led to an enhanced reduction of tumor growth when compared to each reagent alone without induction of toxicity. These observations suggest that L-asparaginase might be useful for the treatment of malignant glial neoplasms.

## INTRODUCTION

Amino acid metabolism might represent an “Achilles heel” in cancer since a number of tumors acquire an altered dependency on some of these metabolic pathways [1-3]. For instance, it has been shown that some cancer cells are particularly dependent on glutamine. As a consequence, glutamine starvation elicits Bax/Bak-dependent apoptosis [4]. Aside from glutamine, asparagine is pivotal for survival of a number of malignancies. This is exemplified most prominently in the setting of acute lymphoblastic leukemia since in many of these malignant cell clones the levels of the enzyme asparagine synthetase (ASNS), which utilizes aspartate and glutamine as a substrate to produce asparagine are suppressed [5, 6]. In turn, asparagine appears to oppose apoptotic cell death, enabling cancer cells to entertain growth. Consequently, pharmaceutical formulations of L-asparaginase are an integral part of combination therapies for ALL. While initially most patients are susceptible, resistance emerges due to several factors, including up-regulation of ASNS [7, 8].

With regards to solid malignancies, L-asparaginase is not commonly employed due to the fact that most solid tumors display relatively high levels of ASNS and are therefore primarily resistant [9, 10]. Nevertheless, there is growing evidence that this view is too simplistic [11] and recent research shows that L-asparaginase might be useful for the treatment of solid malignancies as well [12]. In our study, we focused on glioblastoma WHO IV, which is the most common primary brain tumor in adults and bears a grim prognosis with median survival rates of less than 1.5 years [13]. The current standard of care is mostly focused on chemotherapy and radiation [13]. Most recently, anti-VEGF therapies have gained attention as they prolong progression-free survival, but not overall survival. However, reanalysis of the clinical trial data has shown that patients with glioblastomas of the proneural type displayed a significant increase in overall survival [14], indicating the need for tailored treatment approaches in the sense of precision medicine.

In order to establish a potential new therapeutic strategy for patients with glioblastoma, we analyzed different glioma cell cultures, including established, stem cell-like and patient-derived xenograft (PDX) cells, for their susceptibility to *E. coli*-derived L-asparaginase. Our results suggest that certain glioma cells are particularly sensitive to L-asparaginase. Moreover, we demonstrate that L-asparaginase treatment overcomes resistance to intrinsic and extrinsic apoptosis and therefore may be applicable in the context of combination therapies. Finally, our results suggest that the combination therapy of a BH3-mimetic along with L-asparaginase exerts anti-glioma activity *in vivo*.

## RESULTS

### Treatment with L-asparaginase yields anti-proliferative activity in glioblastoma cells

**Figure 1:**
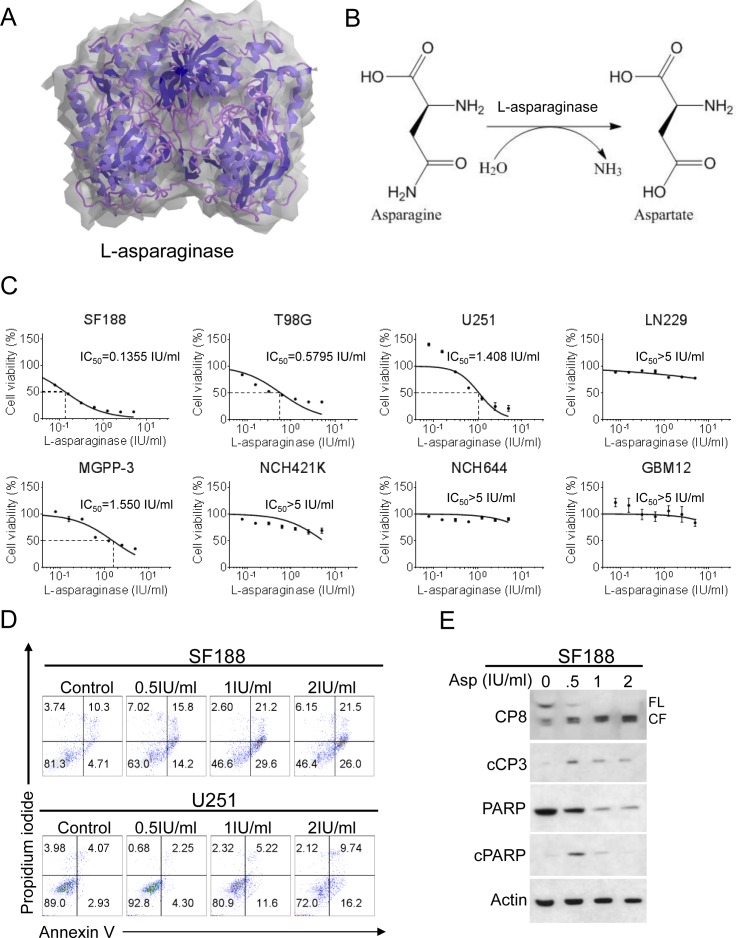
Treatment with L-asparaginase (Asp) inhibits proliferation and induces apoptosis across different glioblastoma cells **A.**, 3-dimensional graphical representation of Escherichia coli L-asparaginase. Modified with ChemBioDraw Ultra 13.0 based on PDB ID 3ECA [57], http://www.rcsb.org/pdb/explore/explore.do?structureId=3eca, last accessed 02/03/2016. **B.**, Representation of the chemical reaction catalyzed by L-asparaginase (ChemBioDraw Ultra 13.0). **C.**, SF188 (pediatric), T98G (adult), U251 (adult), LN229 (adult), MGPP-3 (murine, transgenically-derived) glioblastoma cells and NCH421K, NCH644 glioma stem-like cells as well as GBM12 (PDX-derived) glioblastoma cells were treated with increasing concentrations of L-asparaginase under serum starvation (1.5% FBS). After 72h, MTT assays were performed. Dose-response curves and IC_50_-values were calculated using non-linear regression. Data are presented as mean and SEM. **D.**, Representative flow plots of SF188 and U251 glioblastoma cells subjected to 48h treatment with indicated concentrations of L-asparaginase prior to performing staining for annexin V and propidium iodide. **E.**, SF188 glioblastoma cells were treated for 24h with increasing concentrations of L-asparaginase (Asp) under serum starvation (1.5% FBS). Whole-cell extracts were examined by Western blot for caspase 8 (CP8 - FL = full length form, CF = cleaved fragment), cleaved caspase 3 (cCP3), PARP and cleaved PARP (cPARP). Actin Western blot analysis was performed to confirm equal protein loading.

We first assessed whether treatment with L-asparaginase (Figure 1A and 1B) has anti-proliferative activity in glioblastoma cells *in vitro*. Established glioblastoma cells (SF188, T98G, U251, LN229), glioma stem-like cells (NCH421K, NCH644) and glioblastoma cells derived from a PDX-model (GBM12) or a murine transgenic model (MGPP-3; *PDGFR*+, *PTEN*−/−, *TP53*−/−) were treated for 72h with increasing concentrations of L-asparaginase prior to performing MTT- or CellTiter-Glo^®^-assays. As shown in Figure 1C, treatment with L-asparaginase lead to a dose-dependent anti-proliferative effect on SF188, T98G, U251 and MGPP-3 glioblastoma cells. In LN229 established glioblastoma, NCH421K, NCH644 glioma stem-like and GBM12 (PDX-derived) glioblastoma cells treatment with L-asparaginase yielded only little anti-proliferative activity.

### Treatment with L-asparaginase induces apoptosis in glioblastoma cells

To assess the mechanism of the anti-proliferative effect of L-asparaginase, we treated SF188 and U251 glioblastoma cells with L-asparaginase prior to staining with annexin V and propidium iodide. In both cell lines, treatment with L-asparaginase lead to an enhanced fraction of annexin V-positive cells (apoptotic cells) in a dose-dependent manner (Figure 1D). Consistent with this finding, cleavage of caspases 8 and 3 as well as of PARP was enhanced after treatment with L-asparaginase (Figure 1E). Moreover, when SF188 cells were treated with L-asparaginase and the pan-caspase inihibitor zVAD.fmk combined, the fraction of annexin V-positive cells was markedly reduced compared to cells treated with L-asparaginase alone (Supplementary Figure 1).

### L-asparaginase treatment reduces the mitochondrial membrane potential

**Figure 2:**
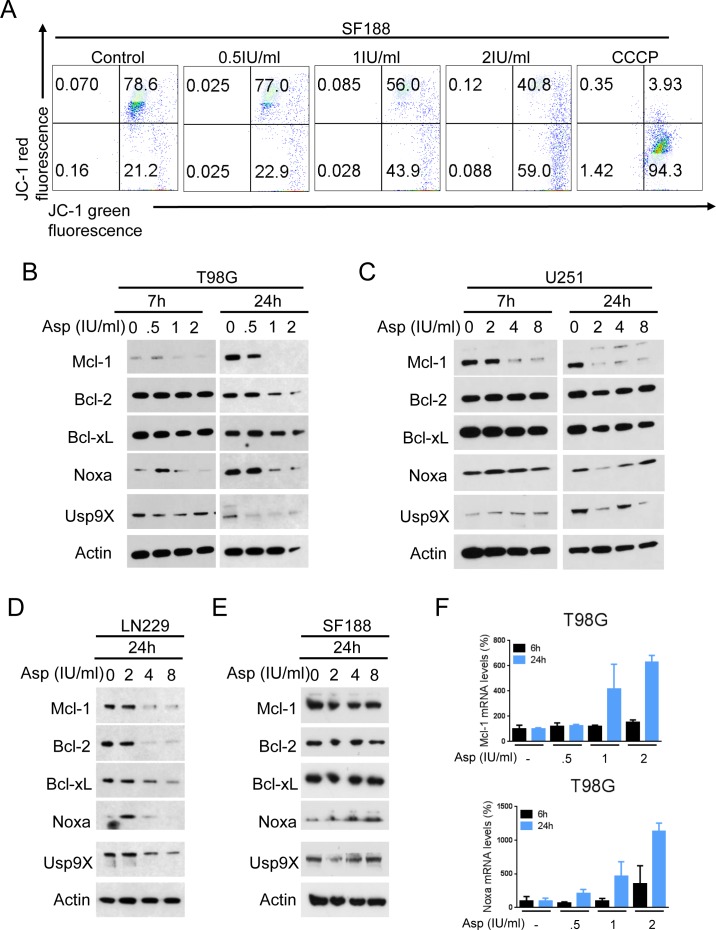
L-asparaginase (Asp) treatment reduces the mitochondrial membrane potential and expression of anti-apoptotic Bcl-2 family proteins **A.**, Representative flow plots of SF188 glioblastoma cells treated with increasing concentrations of L-asparaginase prior to staining for JC-1 and flow cytometric analysis. Treatment with the mitochondrial uncoupler carbonyl cyanide 3-chlorophenylhydrazone (CCCP) served as positive control. **B**.-**E**., T98G (**B**), U251 (**C**), LN229 (**D**) and SF188 (**E**) glioblastoma cells were treated as indicated with L-asparaginase under serum starvation (1.5% FBS). Whole-cell extracts were examined by Western blot for Mcl-1, Bcl-2, Bcl-xL, Noxa and Usp9X. Actin served as a loading control. **F.**, T98G glioblastoma cells were treated for 6 or 24h with increasing concentrations of L-asparaginase prior to performing rtPCR for Mcl-1 and Noxa. Columns, mean. Bars, SD.

Since L-asparaginase induced apoptosis in glioblastoma cells, we next examined whether this observation is at least in part due to activation of intrinsic apoptosis. We therefore performed staining for JC-1 in SF188 glioblastoma cells treated with increasing concentrations of L-asparaginase. As shown in Figure 2A, treatment with L-asparaginase yielded a marked reduction of the mitochondrial membrane potential, suggesting a mitochondrial component of the apoptotic response.

### Treatment with L-asparaginase leads to down-regulation of Mcl-1

Our observations so far pointed towards an apoptotic response that is at least in part mitochondrially-driven. Therefore, we focused next on effects of L-asparaginase on the expression of anti-apoptotic Bcl-2 family members. Treatment with L-asparaginase resulted in a marked down-regulation of Mcl-1 in T98G, U251 and LN229 glioblastoma cells (Figure 2B-2D). Expression of Bcl-2 was reduced in T98G and LN229 glioblastoma cells. Bcl-xL expression was only reduced in LN229 cells. In SF188 glioblastoma cells, none of the anti-apoptotic Bcl-2 family members we examined were down-regulated under these conditions (Figure 2E). However, protein levels of the pro-apoptotic Mcl-1-specific BH3-only protein Noxa were markedly increased after treatment with L-asparaginase in SF188 cells - a finding also observed at lower concentrations of L-asparaginase in T98G and LN229 glioblastoma cells, indicating a potential cell type-specific response to L-asparaginase treatment.

### L-asparaginase-mediated down-regulation of Mcl-1 is due to a post-transcriptional mechanism

To further assess by which mechanism Mcl-1 is down-regulated, we performed real-time PCR analysis in T98G glioblastoma cells. Treatment with L-asparaginase did not result in reduced Mcl-1 mRNA expression after 6h, but even in a markedly enhanced expression after 24h indicating a post-transcriptional mechanism (Figure 2F).

### Up-regulation of Noxa is transcriptionally mediated

We next assessed by which mechanism Noxa is up-regulated. Real-time PCR analysis in T98G glioblastoma cells showed that treatment with L-asparaginase resulted in increased Noxa mRNA expression after 6h which was even more pronounced after 24h suggesting at least in part a transcriptional mechanism (Figure 2F). Since Noxa modulation was observed in cells that are *TP53*-mutated, it appears likely that L-asparaginase-mediated up-regulation of Noxa is independent of p53 signaling.

### Treatment with L-asparaginase sensitizes for intrinsic apoptosis

**Figure 3:**
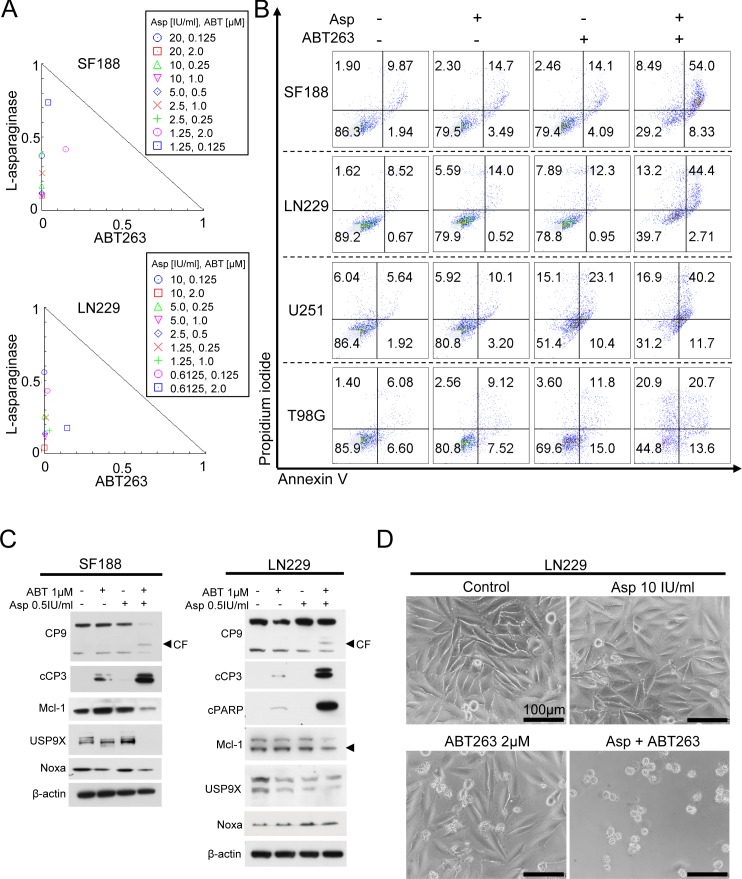
Combined treatment with L-asparaginase (Asp) and ABT263 results in anti-proliferative and pro-apoptotic synergism **A.**, SF188 and LN299 glioblastoma cells were treated for 72h with L-asparaginase and ABT263 as indicated prior to performing MTT-assays. Normalized isobolograms were calculated using the CompuSyn software. The connecting line represents additivity. Data points located below the line indicate a synergistic drug-drug interaction and data points above the line indicate an antagonistic drug-drug interaction. **B.**, Representative flow plots of SF188 (Asp 0.5IU/ml, ABT263 1μM, 24h), LN229 (Asp 2IU/ml, ABT263 0.5μM, 24h), U251 (Asp 2IU/ml, ABT263 1.5μM, 24h) and T98G (Asp 1IU/ml, ABT263 1μM, 48h) glioblastoma cells subjected to treatment with L-asparaginase, ABT263 or the combination prior to staining for annexin V/propidium iodide and flow cytometric analysis. **C.**, SF188 and LN229 glioblastoma cells were treated with L-asparaginase and/or ABT263 as indicated. Western blot analysis was performed for caspase 9 (CP9), cleaved caspase 3 (cCP3), cleaved PARP (cPARP), Mcl-1, Usp9X and Noxa. Actin expression was determined to confirm equal protein loading. CF = cleaved fragment; arrow head represents specific band for Mcl-1. **D.**, Representative microphotographs of LN229 glioblastoma cells treated with 10 IU/ml L-asparaginase, 2 μM ABT263 or the combination for 48h. Magnification, x40; scale bar, 100 μm.

Enhanced expression or function of Mcl-1 has been shown to represent a major mediator of resistance towards ABT compounds such as ABT263. Given that treatment with L-asparaginase resulted in either down-regulation of Mcl-1 or up-regulation of its pro-apoptotic counterpart, Noxa, we next assessed whether this molecular observation would sensitize for treatment with ABT263. As shown in Figure 3A, combined treatment with L-asparaginase and ABT263 yielded a synergistic anti-proliferative effect across a wide range of different pairs of concentrations tested in SF188, LN229, T98G, U251 glioblastoma cells and NCH644 glioma stem-like cells (Figure 3A, Table 1 and Supplementary Figure 2). These observations are mirrored by marked morphological changes as shown in representative microphotographs in LN229 cells (Figure 3D). To assess the mechanism of this effect we performed staining for annexin V/PI in SF188, LN229, U251 and T98G glioblastoma cells after treatment with the combination or single agents (Figure 3B). Consistently, combined treatment with L-asparaginase and ABT263 resulted in at least additive pro-apoptotic effects across all cell lines tested. On the molecular level, combined treatment resulted in enhanced cleavage of caspases 9 and 3 in SF188 and LN229 as well as of PARP in LN229 glioblastoma cells (Figure 3C). Consistent with these observations, protein levels of Mcl-1 and its deubiquitinase Usp9X were markedly decreased in SF188 glioblastoma cells treated with the combination. In LN229 glioblastoma cells, this effect was less pronounced. However, expression of the pro-apoptotic counterpart of Mcl-1, Noxa, was markedly enhanced.

**Table 1:** Combined treatment with ABT263 and L-asparaginase (Asp) results in a synergistic anti-proliferative effect in SF188, LN229 and NCH644 glioblastoma cells

SF188			LN229			NCH644		
Asp (IU/ml)	ABT263 (μM)	CI	Asp (IU/ml)	ABT263 (μM)	CI	Asp (IU/ml)	ABT263 (μM)	CI
20.0	0.125	0.37373	10.0	0.125	0.55783	20.0	0.93	0.17568
20.0	2.0	0.10444	10.0	2.0	0.04420	20.0	15.0	0.28107
10.0	0.25	0.16943	5.0	0.25	0.25976	10.0	1.87	0.39124
10.0	1.0	0.10173	5.0	1.0	0.12358	10.0	7.5	0.38818
5.0	0.5	0.12043	2.5	0.5	0.14549	5.0	3.75	0.51880
2.5	1.0	0.25964	1.25	0.25	0.25930	2.5	7.5	0.53591
2.5	0.25	0.38731	1.25	1.0	0.18630	2.5	1.87	0.46571
1.25	2.0	0.56838	0.6125	0.125	0.45275	1.25	15.0	0.25492
1.25	0.125	0.77678	0.6125	2.0	0.32081	1.25	0.93	0.35660

The CompuSyn software (ComboSyn, Inc., Paramus, NJ, U.S.A.) was used for the drug-drug interaction analysis including the calculation of the combination index (CI). A CI < 1 was considered as synergistic, a CI = 1 as additive and a CI>1 as antagonistic.

### Treatment with L-asparaginase sensitizes for extrinsic apoptosis

**Figure 4:**
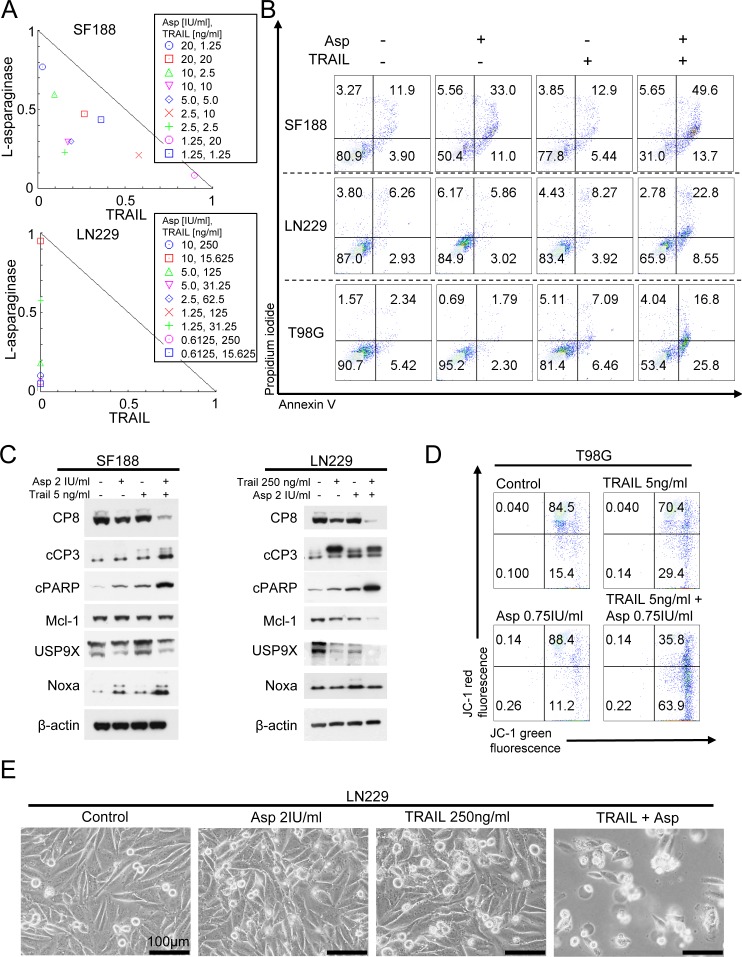
Combined treatment with L-asparaginase (Asp) and TRAIL results in anti-proliferative and pro-apoptotic synergism **A.**, SF188 and LN299 glioblastoma cells were treated for 72h with L-asparaginase and TRAIL as indicated prior to performing MTT-assays. Normalized isobolograms were calculated using the CompuSyn software. The connecting line represents additivity. Data points located below the line indicate a synergistic drug-drug interaction and data points above the line indicate an antagonistic drug-drug interaction. **B.**, Representative flow plots of SF188 (Asp 0.5IU/ml, TRAIL 25 ng/ml), LN229 (Asp IU/ml, TRAIL 100 ng/ml) and T98G (Asp 0.75IU/ml, TRAIL 4ng/ml) glioblastoma cells subjected to treatment with L-asparaginase, TRAIL or the combination for 24h prior to staining for annexin V/propidium iodide and flow cytometric analysis. **C.**, SF188 and LN229 glioblastoma cells were treated with L-asparaginase and/or TRAIL as indicated. Western blot analysis was performed for caspase8 (CP8), cleaved caspase 3 (cCP3), cleaved PARP (cPARP), Mcl-1, Usp9X and Noxa. Actin expression was determined to confirm equal protein loading. **D.**, Representative flow plots of T98G glioblastoma cells treated with increasing concentrations of L-asparaginase prior to staining for JC-1 and flow cytometric analysis. E, Representative microphotographs of LN229 glioblastoma cells treated with 2 IU/ml L-asparaginase, 250 ng/ml TRAIL or the combination for 48h. Magnification, x40; scale bar, 100 μm.

Since we observed that treatment with L-asparaginase sensitizes for mitochondrially driven apoptosis, we next assessed whether L-asparaginase also sensitizes for apoptosis mediated through death-promoting ligands. We therefore treated SF188 and LN229 glioblastoma cells with L-asparaginase, TNFα-related apoptosis-inducing ligand (TRAIL) or the combination of both as indicated. Combined treatment leads to a pronounced synergistic anti-proliferative effect in both cell lines tested (Figure 4A and Table 2). These findings are reflected by prominent changes in morphology as illustrated by respective microphotographs (Figure 4E). To assess the subjacent mechanism, staining for annexin V/PI was performed in SF188, LN229 and T98G glioblastoma cells. Consistent with our previous isobologram analysis, the fraction of annexin V-positive (apoptotic) cells was enhanced in a synergistic manner when L-asparaginase was combined with TRAIL (Figure 4B). On the molecular level, expression of the total form of caspase 8 was significantly decreased in SF188 and LN229 cells treated with the combination, indicating enhanced cleavage of caspase 8 and enhanced activation of the extrinsic pathway (Figure 4C). Consistent with this observation, cleavage of caspase 3 and PARP was increased. Notably, expression of the cleaved form of caspase 3 did not differ substantially when comparing LN229 cells treated with TRAIL alone or the combination. However, total caspase levels (see caspase-8) are already depleted, suggesting faster kinetics of caspase cleavage due to the combination treatment.

**Table 2:** Combined treatment with TRAIL and L-asparaginase (Asp) results in a synergistic anti-proliferative effect in SF188 and LN229 glioblastoma cells

SF188			LN229		
Asp (IU/ml)	TRAIL (ng/ml)	CI	Asp (IU/ml)	TRAIL (ng/ml)	CI
20.0	1.25	0.79743	10.0	250.0	0.10414
20.0	20.0	0.73819	10.0	15.625	0.95175
10.0	2.5	0.69211	5.0	125.0	0.18268
10.0	10.0	0.46664	5.0	31.25	1.06497
5.0	5.0	0.49002	2.5	62.5	1.33575
2.5	10.0	0.79353	1.25	125.0	2.77706
2.5	2.5	0.38581	1.25	31.25	0.57995
1.25	20.0	0.98086	0.6125	250.0	0.06601
1.25	1.25	0.79618	0.6125	15.625	0.05228

To examine whether the pro-apoptotic synergism of the combination treatment is at least in part due to an enhanced activation of the mitochondrial pathway we performed JC-1-staining. As shown in Figure 4D, combined treatment with TRAIL and L-asparaginase lead to a marked loss of the mitochondrial membrane potential. Based on this finding we performed Western blot analysis of Mcl-1 and its interacting proteins Usp9X and Noxa. Combined treatment with L-asparaginase and TRAIL yielded a marked down-regulation of Mcl-1 and Usp9X in LN229 glioblastoma cells (Figure 4C). In contrast, in SF188 glioblastoma cells Mcl-1 expression was not significantly affected by the combination treatment. Moreover, there was no additional down-regulation of Usp9X levels when comparing SF188 cells treated with TRAIL alone or the combination. However, expression of the pro-apoptotic protein Noxa was markedly enhanced following combined treatment with L-asparaginase and ABT263.

### Knock-down of Mcl-1 sensitizes for treatment with ABT263 and TRAIL

**Figure 5:**
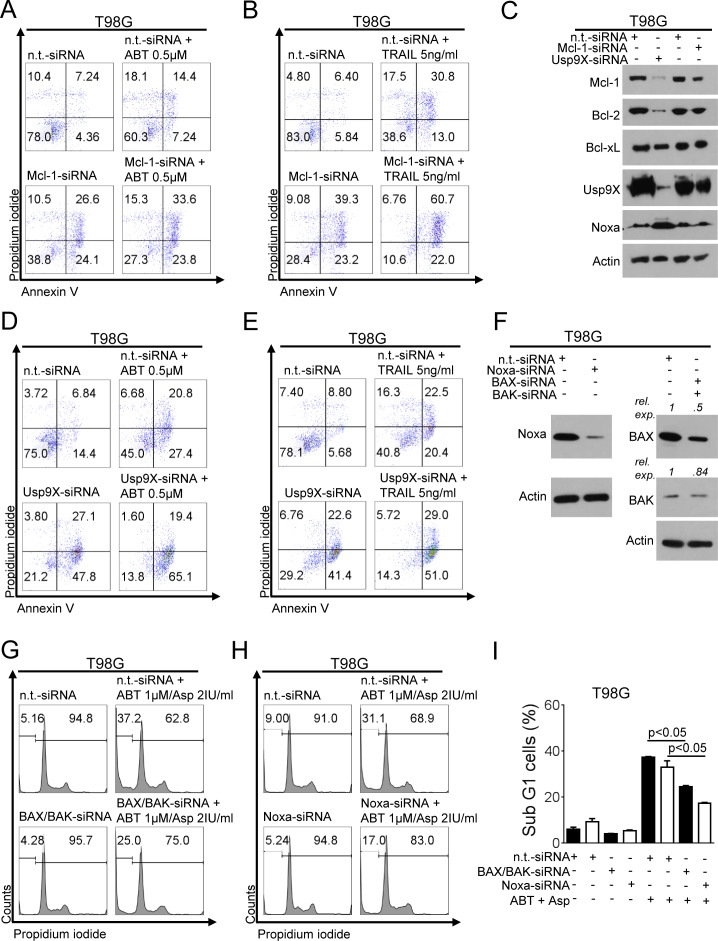
Knock-down for Mcl-1 or Usp9X sensitizes for ABT263- or TRAIL-mediated apoptosis **A.**, T98G glioblastoma cells were treated with non-targeting (n.t.)-siRNA, or Mcl-1-siRNA followed by a treatment with ABT263 or solvent for 24h. Staining for annexin V/propidium iodide (PI) was performed prior to flow cytometric analysis. Representative flow plots are shown. **B.**, T98G glioblastoma cells were treated with non-targeting (n.t.)-siRNA, or Mcl-1-siRNA followed by a treatment with TRAIL or solvent for 24h. Staining for annexin V/PI was performed prior to flow cytometric analysis. Representative flow plots are shown. **C.**, T98G glioblastoma cells were transfected with n.t.-siRNA, Mcl-1-siRNA or Usp9X-siRNA. Whole-cell extracts were collected prior to Western blot analysis for Mcl-1, Bcl-2, Bcl-xL, Usp9X and Noxa. Actin served as a loading control. **D.**, T98G glioblastoma cells were treated with non-targeting (n.t.)-siRNA, or Usp9X-siRNA followed by a treatment with ABT263 or solvent for 24h. Staining for annexin V/PI was performed prior to flow cytometric analysis. Representative flow plots are shown. **E.**, T98G glioblastoma cells were treated with non-targeting (n.t.)-siRNA, or Usp9X-siRNA followed by a treatment with TRAIL or solvent for 24h. Staining for annexin V/PI was performed prior to flow cytometric analysis. Representative flow plots are shown. **F.**, T98G glioblastoma cells were transfected with n.t.-siRNA, Noxa-siRNA BAX-siRNA or BAK-siRNA. Whole-cell extracts were collected prior to Western blot analysis for BAX, BAK and Noxa. Actin served as a loading control. **G.**, T98G glioblastoma cells were treated with non-targeting (n.t.)-siRNA or BAX/BAK-siRNA followed by a treatment with the combination of ABT263 and L-asparaginase (Asp) for 24h. Staining for PI was performed prior to flow cytometric analysis. Representative flow plots are shown. **H.**, T98G glioblastoma cells were treated with non-targeting (n.t.)-siRNA or Noxa-siRNA followed by a treatment with the combination of ABT263 and L-asparaginase (Asp) for 24h. Staining for PI was performed prior to flow cytometric analysis. Representative flow plots are shown. **I.**, Quantitative representation of the fraction of subG1-cells treated as described for G (black columns) and H (white columns with black border). Columns, means. Bars, SD.

Given that L-asparaginase modulated Mcl-1 levels in various glioblastoma cells tested, we examined whether knock-down of Mcl-1 would be sufficient to enhance apoptosis induced by ABT263. SiRNA-mediated knock-down of Mcl-1 enhanced apoptosis induced by ABT263 in T98G glioblastoma cells and thereby phenocopied the sensitizing effect of L-asparaginase on ABT263 (Figure 5A). Since we also observed that combined treatment with L-asparaginase and TRAIL yielded a synergistic pro-apoptotic effect, we tested the hypothesis that Mcl-1 is involved in this process. Akin to ABT263, Mcl-1 knockdown enhanced apoptosis induced by TRAIL (Figure 5B). Knock-down of Mcl-1 was confirmed by Western blot analysis (Figure 5C).

### Knock-down of Usp9X sensitizes for treatment with ABT263 and TRAIL

Since treatment with L-asparaginase leads to a marked down-regulation of Usp9X, we next assessed whether knock-down of Usp9X would mirror the pro-apoptotic synergism of L-asparaginase and ABT263 or TRAIL. We therefore performed knock-down experiments with Usp9X-siRNA. As shown in Figure 5D and 5E, siRNA-mediated knock-down of Usp9X resulted in a markedly enhanced fraction of annexin V-positive cells when combined with ABT263 or TRAIL, suggesting that down-regulation of Usp9X, as seen after L-asparaginase treatment, would suffice to sensitize for both extrinsic and mitochondrially-driven apoptosis. Western blot analysis was performed to verify efficient knock-down of Usp9X (Figure 5C).

### Simultaneous knock-down of Bax and Bak attenuates apoptosis induced by combined treatment with ABT263 and L-asparaginase

Our data showed that L-asparaginase treatment causes a decrease in the mitochondrial membrane potential. We therefore next assessed whether knock-down of the pro-apoptotic multi-domain effector proteins Bax and Bak would decrease the apoptotic response towards combined treatment with ABT263 and L-asparaginase. As shown in Figure 5G and 5I, in T98G cells treated with ABT263 and L-asparaginase combined, knock-down of Bax and Bak yielded a marked reduction of the fraction of sub-G1 cells (apoptotic cells). However, restoration of baseline apoptotic levels was not reached which could be explained by an incomplete knock-down of Bax and or Bak (Figure 5F) or only partial involvement of a mitochondrial pathway in the apoptotic response.

### Knock-down of Noxa reduces ABT263/L-asparaginase-mediated apoptosis

Based on our observation, that treatment with L-asparaginase lead to an up-regulation of Noxa protein levels under certain conditions, we next examined whether Noxa is involved in the pro-apoptotic effect of the combination therapy. As shown in Figure 5H and 5I, combined treatment with L-asparaginase and ABT263 lead to a significantly decreased fraction of sub-G1 cells in T98G cells when Noxa was silenced. Notably, complete rescue was not achieved indicating at the contribution of additional molecular events. Knock-down of Noxa was verified by Western blot (Figure 5F).

### Combined treatment with L-asparaginase and ABT263 results in enhanced inhibition of tumor growth *in vivo*

**Figure 6:**
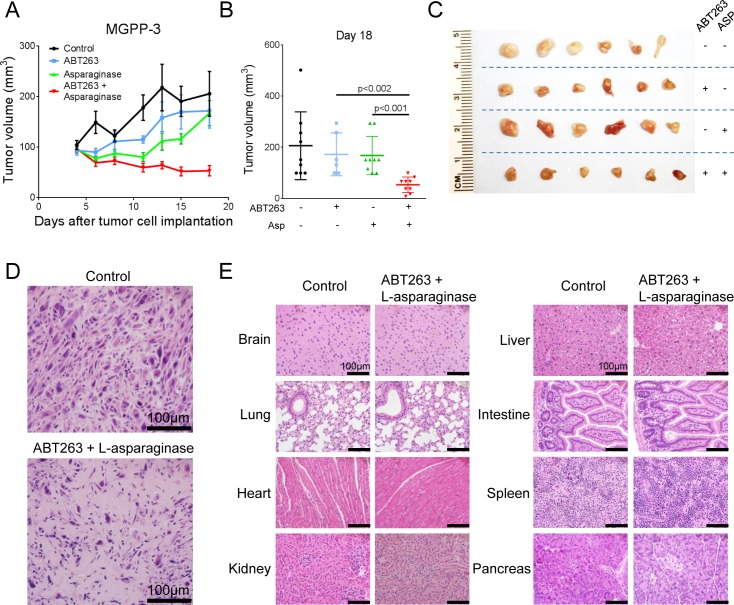
Combined treatment with L-asparaginase (Asp) and ABT263 yields enhanced anti-tumorigenic efficacy *in vivo* 1×10^6^MGPP-3 glioblastoma cells were implanted subcutaneously. After tumor formation animals were treated intraperitoneally with vehicle (*n* = 9 tumors), ABT263 (25 mg/kg; *n* = 9 tumors), L-asparaginase (1500 IU/kg; *n* = 9 tumors) or both agents (*n* = 9 tumors) 3 times/week over 2 weeks. **A.**, Tumor growth curves showing the increase in tumor size for each treatment group. Data are presented as means and SEM. **B.**, Quantification and statistical analysis (Student's *t*-test) of the tumor size among different treatment groups 18 days after tumor implantation. **C.**, Representative photographs of the tumors. **D.**, Representative microphotographs showing the histological morphology (H&E staining) of tumors from animals receiving treatment either with vehicle or the combination of ABT263 and L-asparaginase. Magnification, x40; scale bar, 100μm. **E.**, Representative microphotographs showing the histological morphology (H&E staining) of the indicated organs among animals receiving treatment either with vehicle or the combination of ABT263 and L-asparaginase. Magnification, x40; scale bar, 100μm.

We next assessed whether the combination treatment with L-asparaginase and ABT263 would provide a therapeutic benefit *in vivo*. Therefore, MGPP-3 (*PDGF*+, *PTEN*−/−, *p53*−/−) glioblastoma cells, that were derived from a transgenic proneural mouse model, were implanted subcutaneously into SCID SHO mice. After tumor formation, the mice were randomized and treatment with ABT263 (25 mg/kg), L-asparaginase (1500 IU/kg), both agents or solvent was started. Combined treatment with ABT263 and L-asparaginase resulted in statistically significant smaller tumors when compared to single-agent or vehicle treatments (Figure 6A and 6B). Photographs of representative tumors are provided in Figure 6C and are in accordance with the notion that the combination treatment leads to an enhanced reduction of the tumor growth rate (Figure 6C). Notably, mean tumor size at the end of the experiment was smaller in animals treated with the combination when compared to the tumor size at onset of therapy indicating that this treatment not only reduced the tumor growth rate but even caused a mean regression of tumor size of 45.24% (Figure 6A). This finding is mirrored by a reduced cellularity in tumors harvested from animals subjected to the combination treatment (Figure 6D). To detect potential organotoxic effects histological analysis was performed which showed no tissue alterations in the indicated organs (Figure 6E).

## DISCUSSION

Because of their aggressive growth rate and their inability to undergo physiological cell death, cancer cells acquire a variety of metabolic changes [15]. Most prominently known are the metabolic alterations in the glycolytic pathway, which were initially observed by the German scientist, Otto Warburg, in the last century and referred to as “aerobic glycolysis” [15, 16]. The term “aerobic glycolysis” implies that despite the abundance of oxygen, cancer cells metabolize glucose *via* pyruvate to lactate instead of subjecting pyruvate to oxidative decarboxylation in the pyruvate-dehydrogenase reaction, resulting in the production of acetyl-coA. While intuitively one would anticipate that cancer cells produce energy in the most efficient fashion, aerobic glycolysis is somewhat contradictory to this expectation since in aerobic glycolysis carbon is shunted away from the citric acid cycle and oxidative phosphorylation, resulting in a significant loss in NADH_2_ oxidation and ATP generation. While aerobic glycolysis is inefficient in energy production, it provides cancer cells with the unique opportunity to utilize carbon sources for the generation of nucleotides, enabling efficient DNA synthesis, cell division and an overall anabolic state [16].

Tumor cells have evolved with additional strategies to compensate for this rather inefficient energy production through reliance on amino acid metabolism [17]. In that context, many tumor cells, including glioblastoma cells (e.g. SF188), heavily depend on glutamine [18, 19], which is in part regulated by the oncogene c-myc. In that context, c-myc was shown to regulate several enzymes and transporters that facilitate glutamine metabolism, which is one of the reasons why interference with myc in myc-dependent tumors might be utilized as a therapy. While glutamine may serve as an energy source, it also represents a source for anaplerosis to maintain the activity of the citric acid cycle, which in turn provides metabolites, such as succinyl-coA, that are essential for the synthesis of pivotal cellular molecules [20, 21]. A recent report linked the amino acid asparagine to glutamine metabolism since in glutamine-susceptible tumor cells addition of asparagine rescued from glutamine withdrawal-mediated cell death [4]. This was in part explained by the notion that glutamine is a substrate for asparagine synthetase (ASNS) and consequently loss of glutamine would deplete cells from asparagine. In certain non-solid malignancies, ASNS is expressed at relatively low levels which suggests that such cells may be prone to asparagine depletion caused by L-asparaginase. Indeed, different formulations of L-asparaginase have been an integral part of the treatment for acute lymphoblastic leukemia for many years [22]. Resistance to L-asparaginase treatment is often accompanied by up-regulation of ASNS [10, 11, 23, 24]. Therefore, means to inhibit ASNS enzyme activity or synthesis are considered to be helpful to counteract evolving resistance. In addition, ASNS levels were shown to correlate with unfavorable disease outcome in several malignancies.

Since gliomas akin to other tumors display dependency on amino acids (foremost for driving protein synthesis), glutamine metabolism and potentially consequently on asparagine, we hypothesized that depletion of asparagine might be a potential therapeutic approach for the treatment of gliomas. In order to test this hypothesis, we assessed the effects of *E. coli*-derived L-asparaginase on a number of different glioma cell cultures including patient-derived xenograft (PDX) and stem cell-like glioma cells. Our findings showed that SF188 pediatric glioblastoma cells display a remarkable sensitivity to L-asparaginase in contrast to LN229 GBM cells. These observations are in agreement with earlier reports, showing that LN229 is resistant to glutamine withdrawal-induced cell death, while in contrast SF188 is highly susceptible. In sensitive cells, L-asparaginase-induced apoptosis was accompanied by dissipation of the mitochondrial membrane potential and activation of effector caspases. These findings are in agreement with previous reports in solid and non-solid malignancies [6, 25, 26]. Given the relatively high sensitivity of SF188 to L-asparaginase, it is conceivable that other glioblastomas might be susceptible in a similar manner. For this reason, it is of utmost importance to identify predictive biomarkers. In the era of precision medicine, many interesting new markers have been identified recently, including mutations of histone and IDH1 proteins. Specifically, IDH1-mutated gliomas are a defined group of neoplasms that includes secondary glioblastomas and oligodendrogliomas. Due to the IDH1 mutation these neoplasms gain a neomorphic enzymatic activity, resulting in the production of 2-hydroxyglutarate which appears to modulate many features of IDH1-mutated gliomas including a change in the methylation pattern and dependency on certain pathways of the amino acid metabolism. Future studies may also indicate whether the IDH1 mutation might be a predictive biomarker for certain therapeutics, potentially also entailing L-asparaginase.

Some glioma cell cultures are relatively resistant towards apoptosis induction by L-asparaginase and the underlying mechanisms for this phenomenon remain to be elucidated. One potential explanation might be that L-asparaginase elicits a cytoprotective form of autophagy in glioma cells, which in turn dampens overall cell death. Consistent with this assumption, L-asparaginase caused a form of cytoprotective autophagy in chronic myeloid leukemia (CML) cells and pharmacological interference with L-asparaginase-mediated autophagy (e.g. through the lysosomotropic drug, chloroquine, or the PI3K inhibitor, LY294002) further enhanced L-asparaginase-mediated apoptosis and cell death in model systems of CML [27]. Therefore, targeting cytoprotective autophagy might further enhance cell death induced by L-asparaginase in model systems of glioma and compounds, such as chloroquine or derivatives, might be combined with L-asparaginase.

On the molecular level, L-asparaginase treatment affected the levels of pro- and anti-apoptotic Bcl-2 family members with up-regulation of Noxa and suppression of Mcl-1. Remarkably, cells that revealed relatively little sensitivity to L-asparaginase still showed marked regulation of the Bcl-2 family proteins, suggesting that L-asparaginase might enhance extrinsic as well as intrinsic apoptotic stimuli. In that context, it is worthwhile mentioning that L-asparaginase-mediated down-regulation of anti-apoptotic Bcl-2 family members, such as Mcl-1, does not directly correlate with asparaginase-mediated apoptosis induction, suggesting that the biochemical effects on Bcl-2 family members does not serve as a direct marker for treatment efficacy.

Given that L-asparaginase affects protein synthesis, it is likely that the level of proteins with a short half-life, such as Mcl-1 [28, 29], c-FLIP [30, 31] or survivin [32] are decreased by L-asparaginase which in turn increases TRAIL-mediated apoptosis. Consistently, our findings showed that L-asparaginase broadly sensitized glioblastoma cells to TRAIL-mediated apoptosis in a synergistic fashion. Mechanistically, this was achieved in part by the ability of L-asparaginase to deplete Mcl-1 [33] along with the deubiquitinase Usp9X [34] which is known to stabilize Mcl-1 protein. While recombinant TRAIL as a therapeutic has somewhat fallen short of expectation, TRAIL-signaling cascade is still exploited as a potential means for therapy [35-38].

This is reflected by the development of antibodies which agonistically activate TRAIL-receptor 1 and 2 and more recently by a small molecule that increases both the levels of TRAIL as well as of death receptors. While this molecule, TIC10/ONC201, was initially inaugurated in 2013, it is remarkable that this molecule has entered phase II clinical trials in patients [39-41]. While recombinant TRAIL has never reached clinical trials for the treatment of glioblastoma, TIC10/ONC201 is being studied in patients suffering from recurrent high-grade gliomas (https://clinicaltrials.gov/ct2/show/NCT02525692?term=NCT02525692&rank=1, last accessed 02/08/2016). Recombinant TRAIL is not ideal with regards to its pharmacokinetics, e.g. its high susceptibility to degradation in the plasma. In contrast, TIC10/ONC201 as a small molecule possesses many advantages, including more suitable pharmacokinetics. Given its ability to inhibit other factors that are involved in TRAIL resistance, TIC10/ONC201 might serve both as an inducer of TRAIL as well as a sensitizer [41].

In connection with intrinsic apoptosis, L-asparaginase broadly enhanced ABT263-mediated apoptosis in a synergistic fashion even in L-asparaginase-resistant cells such as the LN229 and stem cell-like glioma cell culture NCH644. Our observations suggest that L-asparaginase sensitizes glioma cells to ABT263 in part by modulation of Noxa, Mcl-1 and Usp9X levels. The finding that L-asparaginase decreases levels of the deubiquitinase Usp9X has not been observed thus far. In contrast, inhibition of Mcl-1 expression was shown in other tumor entities before [25]. While Noxa was increased both at the level of mRNA and protein, Mcl-1 was only decreased at the protein level, suggesting L-asparaginase-mediated suppression of Mcl-1 occurs most likely independent of transcription. Results by others have suggested that L-asparaginase potently inhibits mTORC1 signaling and thereby interferes with protein synthesis in cancer cells [42]. Consequently, proteins with a short half-life, e.g. Mcl-1 or the inhibitor of apoptosis protein, survivin, are rapidly depleted [43]. Despite the inhibitory effect on protein synthesis, L-asparaginase may control the stability of Mcl-1 as well since Mcl-1 was shown to be highly prone to proteasomal degradation [44]. Factors that might mediate a transcriptional increase of Noxa are ATF4 and p53 [45, 46]. Since Noxa levels were up-regulated in *TP53-*mutated cells, it is more likely that Noxa levels were increased independent of *TP53*.

Finally, we show that L-asparaginase along with the Bcl-2/Bcl-xL inhibitor ABT263 reduced tumor growth *in vivo* more efficiently than each compound on its own without induction of significant toxicity, providing a proof of concept that glioblastoma cells are more susceptible to treatments involving L-asparaginase. Future studies need to show whether L-asparaginase treatments are also effective in orthotopic model systems of glioblastoma. Since L-asparaginase mainly acts by depletion of L-asparagine from the extracellular environment, the issue of overcoming the blood brain barrier may not be critical for this compound.

## MATERIALS AND METHODS

### Ethics statement

All procedures were in accordance with Animal Welfare Regulations and approved by the Institutional Animal Care and Use Committee at the Columbia University Medical Center.

### Reagents

Recombinant L-asparaginase from Escherichia coli was purchased from Sigma Aldrich (St. Louis, MO, U.S.A.) or from ProSpec-Tany Techno Gene Ltd. (Rehovot, Israel). A 500 IU/ml working solution in PBS was prepared prior to storage at −20°C. ABT263 was purchased from ChemieTek (Indianapolis, IN, U.S.A.). A 10 mM working solution in dimethylsulfoxide (DMSO) was prepared prior to storage at −20°C. Recombinant TRAIL was purchased from Peprotech (Rocky Hill, NJ, U.S.A.). A 100 μg/ml working solution in PBS was prepared prior to storage at −20°C.

### Cell cultures and growth conditions

LN229 (*TP53* mut, *PTEN* wt) and T98G (*TP53* mut, *PTEN* mut) [47] human glioblastoma cells were obtained from the American Type Culture Collection (Manassas, VA, U.S.A.). U251 (*TP53* mut, *PTEN* mut) glioblastoma cells were kindly provided by Dr. James Goldman (Columbia University, New York, NY, U.S.A.). NCH644 and NCH421K stem cell-like glioma cells were obtained from Cell Line Services (CLS, Heidelberg, Germany). SF188 (*TP53* mut, *PTEN* wt) [47] pediatric glioblastoma cells were kindly provided by Dr. Craig Thompson (Memorial Sloan Kettering Cancer Center, New York, NY, U.S.A.). MGPP-3 *PDGF*+, *p53*−/−, *PTEN*−/−) is a murine proneural glioblastoma cell which was kindly provided by Dr. Peter Canoll (Columbia University, New York, NY, U.S.A.) [48]. GBM12 (*TP53* mut, *PTEN* wt) human, patient-derived glioblastoma primary cultures originated from Dr. Jann Sarkaria (Mayo Clinic, Rochester, MI, U.S.A.). The identities of the glioblastoma cell lines we purchased were confirmed by the respective source of purchase. All cells were cultured as previously described [49]. Briefly, LN229, U251, T98G and MGPP-3 cells were cultured in DMEM with 10% FBS, 4.5g/L glucose, 4mM L-glutamine, 1mM pyruvate, 100 units/ml penicillin and 100 μg/ml streptomycin for maintenance. For experimental conditions these cells were cultured in DMEM containing only 1.5% FBS to mimick the nutrition-starved environment within tumors. For the culture of SF188 the fore-mentioned medium was supplemented in addition with 2mM L-alanyl-L-glutamine (GlutaMAX^TM^-I, Gibco, Japan). NCH644 and NCH421K glioma stem-like cells were cultured in MG-43 medium (CLS, Heidelberg, Germany) for both maintenance and experiments. GBM12 cells were cultured as described before [50].

### Cell viability assays

In order to examine cellular proliferation, 3-[4, 5-dimethylthiazol-2-yl]-2, 5-diphenyltetrazolium bromide (MTT) assays were performed as previously described for adherent cells [51, 52]. Anti-proliferative effects on neurosphere cultures were examined by using the CellTiter-Glo^®^(Promega, Madison, WI) luminescent cell viability assay according to the manufacturer's instructions. Briefly, the assay was performed in 96-well plates. 100μl of CellTiter-Glo^®^Reagent was added to each well containing 100μl medium and cells. Cell lysis was induced by shaking for 2 min on an orbital shaker. Then cells were incubated for 10 min at RT for stabilization of the signal prior to measuring luminescence.

### Measurement of apoptosis and mitochondrial membrane potential

For annexin V/propidium iodide (PI) staining the FITC Annexin V Apoptosis Detection Kit I (BD Pharmingen, San Diego, CA) was used according to the manufacturer's instructions. Staining for PI was performed as previously described [51]. The data were analysed with the FlowJo software (version 8.7.1; Tree Star, Ashland, OR, U.S.A.).

### Real-time PCR and cDNA synthesis

RT-PCR was performed as described before (Pareja et al.) using the following primers: Usp9X forward: GTG TCA GTT CGT CTT GCT CAG C; Usp9X reverse: GCT GTA ACG ACC CAC ATC CTG A; Mcl-1 forward: CCA AGA AAG CTG CAT CGA ACC AT; Mcl-1 reverse: CAG CAC ATT CCT GAT GCC ACC T; GAPDH forward: GTC TCC TCT GAC TTC AAC AGC G and GAPDH reverse: ACC ACC CTG TTG CTG TAG CCA A.

### Western blot analysis

Specific protein expression in cell lines was determined by Western blot analysis as described before [53] using the following primary antibodies: Mcl-1 (1:500; CST: Cell Signaling Technology, Danvers, MA), human caspase-9 (1:1,000; CST), human caspase 8 (1:500; CST), cleaved caspase 3 (1:250; CST), cleaved PARP (Asp214, 1:1000; CST), Bax (1:500; CST), Bak (1:500; CST), Bcl-xL (1:500; CST), Usp9X (1:1000; CST), Noxa (1:500, clone 114C307; Calbiochem), β-actin (1:2,000, clone AC15; Sigma Aldrich) and secondary HRP-linked antibodies were purchased from Santa Cruz Biotechnology Inc. (Santa Cruz, CA).

### siRNA transfection

SignalSilence^®^Usp9X siRNA I #6308 was purchased from CST. Non-targeting siRNA-pool (ON-TARGETplus Non-targeting Pool, # D-001810-10-05), BAX (SMARTpool: ON-TARGETplus BAX siRNA, # L-003308-01) and Mcl-1 (SMARTpool: ON-TARGETplus Mcl-1 siRNA, L-004501-00-0005) were purchased from Thermo Fisher Scientific. PMAIP1 siRNA and BAK siRNA were purchased from Ambion. Transfections were performed as previously described [54, 55]. Briefly, cells were incubated for 6h with the formed complexes of Lipofectamine^®^2000 (Invitrogen, Carlsbad, CA) and the respective siRNA (12-well condition) in DMEM without FBS and antibiotics. After 6h, FBS was added to a total concentration of 1.5%.

### Subcutaneous xenograft model

1 × 10^6^MGPP-3 proneural glioblastoma cells (*PDGF+*, *PTEN−/−*, *TP53−/−*) suspended 1:1 in Matrigel^®^Matrix (Corning Inc., Corning, NY) were implanted subcutaneously into the flanks of 6-8 week-old SCID SHO mice as previously described [53]. Treatment was performed intraperitoneally 3 times a week for 2 weeks. For intraperitoneal application ABT263 was dissolved in 80% Cremophor EL (SIGMA, St. Louis, MO) and 20% Ethanol (Pharmco-Aaper, Brookfield, CT) (v/v). L-asparaginase was dissolved in PBS.

### Histological analysis

Subcutaneous tumors and samples from organs were extracted from SCID SHO mice and fixed for at least 24h in 10% PBS-buffered formalin [56]. Then tissues were embedded in paraffin and 4μm thick sections were cut prior to staining with hematoxylin and eosin. Microphotographs were taken at x40 magnification.

### Statistical analysis

Statistical significance was assessed by Student's *t*-test using Prism version 5.04 (GraphPad, La Jolla, CA). A *p* ≤ 0.05 was considered statistically significant. The CompuSyn software (ComboSyn, Inc., Paramus, NJ - www.combosyn.com last accessed 06/01/15) was used for the drug combination analysis including the calculation of the combination index (CI) and isobologram as described before [58]. A CI < 1 was considered as synergistic, a CI = 1 as additive and a CI > 1 as antagonistic. The concentration for each compound resulting in 50 % inhibition (ED_50_) is normalized to 1, plotted on x- or y-axis and connected by a line which represents the ED_50_ isobologram. Data points of drug combinations plotted below the connecting line represent a synergistic interaction, data points located on the line represent an additive interaction and data points located above the connecting line represent an antagonistic interaction.

## SUPPLEMENTARY MATERIAL FIGURES


