# Supplementary material for: Metabolic reprogramming of glioblastoma cells by L-asparaginase sensitizes for apoptosis in vitro and in vivo
Source: Oncotarget. 2016 May 9;7(23):33512–28. doi: 10.18632/oncotarget.9257 (PMC5085099; doi:10.18632/oncotarget.9257)
Supplement: Supplementary file 1 [file oncotarget-07-33512-s001.pdf]

Metabolic reprogramming of glioblastoma cells by L-asparaginase sensitizes for apoptosis *in vitro* and *in vivo*

Supplementary Material

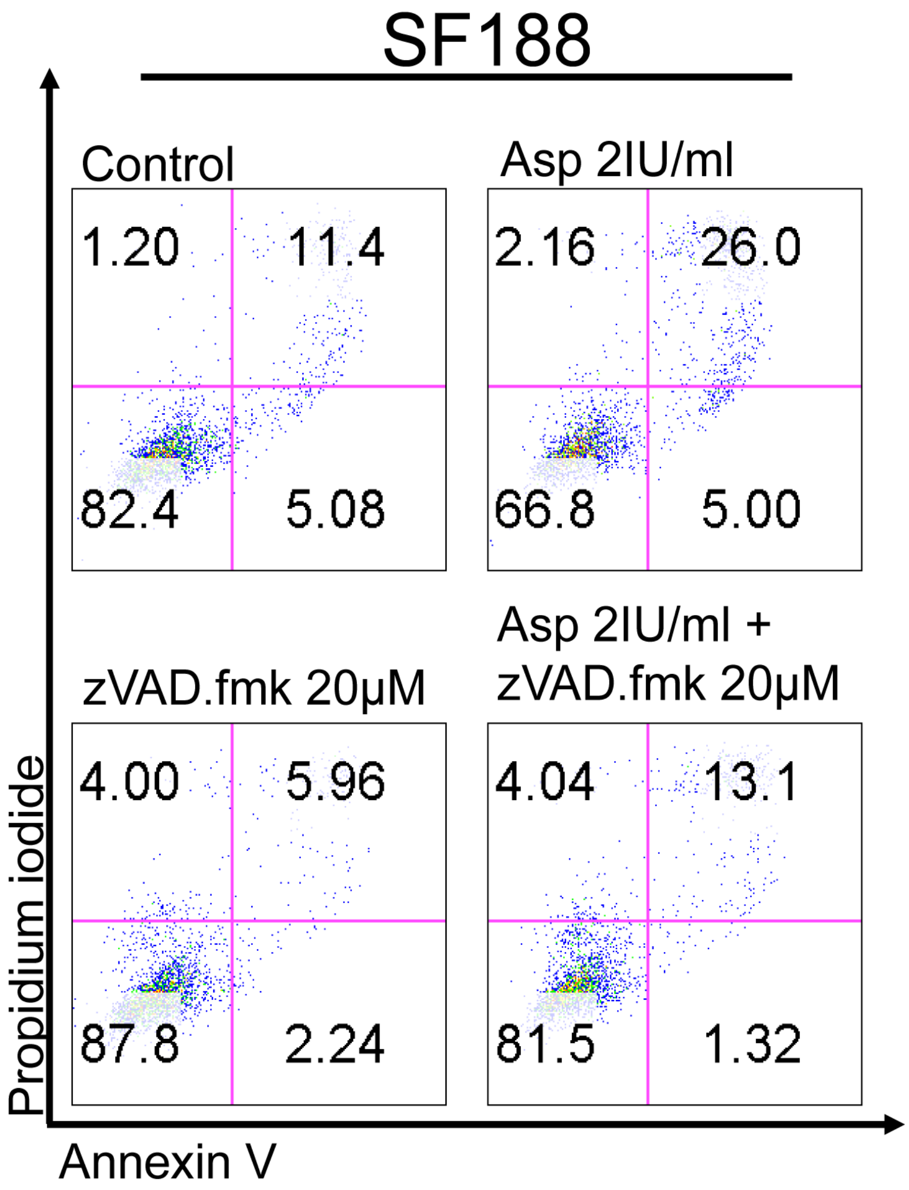

Suppl. fig. 1:

SF188 glioblastoma cells were treated for 24h with L-asparaginase in the absence or presence of the pan-caspase inhibitor zVAD.fmk (20 μM) prior to performing staining for annexin V/propidium iodide and flow cytometric analysis. Representative flow plots are shown.

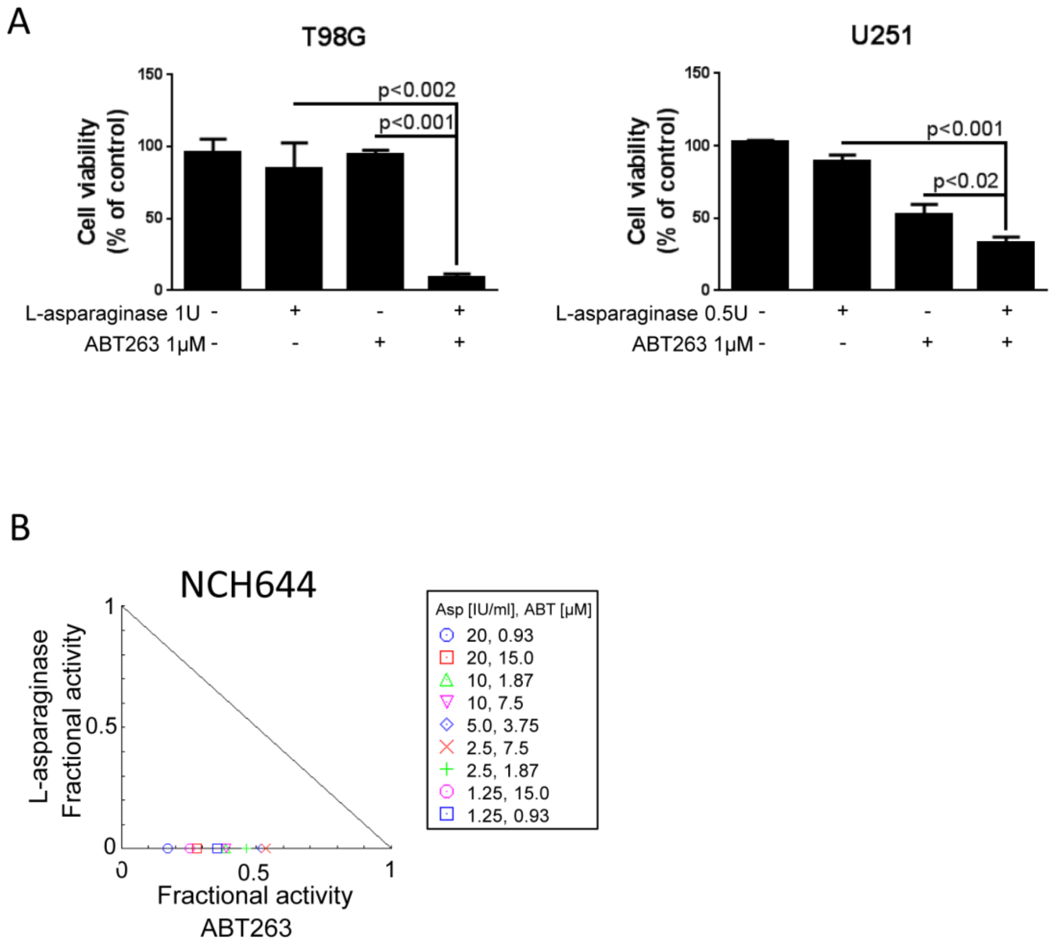

Suppl. fig. 2:

A, T98G and U251 glioblastoma cells were treated for 72h with solvent, ABT263 and/or L-asparaginase as indicated. Afterwards MTT-assays were perfomed to detect anti-proliferative activity. Columns: means. Bars: SD. B, NCH644 glioma stem-like cells were treated for 72h with solvent, ABT263 and/or L-asparaginase as indicated prior to performing CellTiter-Glo® assays. Normalized isobolograms were calculated using the CompuSyn software. The connecting line represents additivity. Data points located below the line indicate a synergistic drug-drug interaction and data points above the line indicate an antagonistic drug-drug interaction.
